# Foreign Body in Orbit

**Published:** 1875-07

**Authors:** M. G. Sloan

**Affiliations:** Delmar, Ia.


Article VII.

### CASE OF FOREIGN BODY IN ORBIT.

By M. G. SLOAN, M.D., Delmar, Ia.

The history of this case is given for publication both on account of its comparative rarity, and with a desire to have the statistics of this class of cases kept as complete as possible.

A. W., aged 23; married; previous health very fair. Met with the below described injury December 14th, 1874, at about 7 P. M. Saw him about three hours afterward. The history of the injury is as follows: In leading a horse through a pair of bars he stumbled and fell prone on the ground, which was thickly studded with weed stubble. He stated that he was conscious of having received some sort of an injury, but could hardly tell, at the time, what its nature was. He walked and crawled to his house, a distance of five or six rods.

On my arrival, found the patient sensible, and suffering very acutely with pain, which extended over the whole head. An incised wound, about five-sixteenths of an inch in length, was visible just above the inner canthus of the right eye. There had been only slight hæmorrhage from the wound. Pulse 60, full and regular. Patient had had a severe chill about an hour before my arrival, and had vomited twice.

On probing the wound, a foreign body was discovered at a depth of about three-quarters of an inch. This was seized with the forceps, and slowly and carefully extracted. It proved to be a part of the circumference of an ordinary weed. Another piece was then laid hold of, and with difficulty extracted. These two pieces, when laid together, almost completed the circumference of the weed, and each piece was of about the same length, viz., nearly one inch. The ends which had looked toward the brain were of such an appearance as to make it seem probable that they had been broken from another segment of weed, which was still imbedded deep in the orbit, or, more likely, in the brain. However, after careful probing, nothing more could be discovered Cold compresses were ordered to head and eye. A purgative dose of sulphate of magnesia given. Warmth applied to extremities. A full dose of morphia administered, and gr. xxx of bromide of potassium ordered to be given every three hours. Perfect quietude enjoined on friends.

On the next day, the 15th, the patient was visited by my partner, Dr. O. E. Deeds, and myself. (We both attended the case to the end.) Pulse about the same as on previous day. Respiration normal; still complaining of severe pain all over the head, and a sense of fullness and throbbing about the orbit.

I have full notes of this case, but will not occupy your space with them.

Symptoms of cephalic inflammation gradually came on.

On the fourth day after the injury, another small piece of weed was discovered in the wound and extracted. It just completed the circumference of the segment of weed already removed. The diameter of the foreign body was five-sixteenths of an inch.

At this time some delirium was observable, and a tendency to coma. There was slight exophthalmos.

About this time, Dr. G. W. Van Zant, of Charlotte, saw the case with my partner and myself, in consultation. He recommended that the present course of treatment be continued, and gave an unfavorable prognosis.

On the fifth day two more pieces of weed were seized and withdrawn. The inner ends of these showed the smooth cut made by the scythe, in mowing down the weeds. These two pieces, when placed together, formed about one-half the circumference of the deeper segment of the weed. The remainder could be felt with the probe, but was too far from the surface to be reached safely. The probe was introduced by the side of this to a depth of two and a half inches, and met with no obstruction. By measurement on a skull, we found that a stick of that length introduced into the orbit would necessarily penetrate the brain.

On the seventh day after the injury, Dr. A. W. Morgan, of DeWitt, was present in consultation. He recommended no change in the course we were pursuing in the case, and also gave an unfavorable prognosis. The patient remained in a comatose condition from this time until December 29th, or the fifteenth day, when we succeeded in removing the remainder of the foreign substance. After this, the coma increased; there was carphologia, involuntary discharges, and muttering delirium. Death occurred January 1st, 1875. The wound discharged pus freely from the first. Stimulants, beef tea, etc., were given as soon as the symptoms indicated failure of the vital powers.

Post mortem refused.

